# Recognition of Facial Expressions in Emotional Situations among Individuals with Mild and Moderate Alzheimer's Disease

**DOI:** 10.1002/alz70857_099702

**Published:** 2025-12-24

**Authors:** Marcia C. N. Dourado, Michelle Brandt, Tatiana Belfort, Marcela Moreira Lima Nogueira

**Affiliations:** ^1^ Federal University of Rio de Janeiro, Rio de Janeiro, Rio de Janeiro, Brazil

## Abstract

**Background:**

Recognizing and processing emotional situations is a critical aspect of social cognition. However, this ability may be impaired in individuals with Alzheimer's disease (AD), which could affect their capacity to navigate social interactions effectively. Emotional recognition deficits in AD are thought to stem from cognitive decline and neural changes in regions associated with emotional and social processing. This study aimed to investigate differences in the comprehension and processing of emotional situations between healthy older controls (HOC) and individuals with varying degrees of AD severity, specifically those with mild (CDR 1) and moderate (CDR 2) AD.

**Method:**

The study employed a cross‐sectional design to assess emotional processing across groups. A convenience sample of 115 participants was divided based on their CDR scores (0, 1, and 2). Participants were evaluated in three contexts: (1) understanding the emotional situation, (2) naming the emotion congruent with the scenario, and (3) choosing the appropriate facial expression from options corresponding to four emotional states—sadness, surprise, anger, and happiness.

**Result:**

The study revealed that the ability to understand emotional contexts, name associated emotions, and choose appropriate facial expressions was not uniformly impaired but depended on the specific emotion being portrayed. For example, emotions such as sadness and anger posed greater challenges for participants with AD, while happiness was more accurately identified across all groups. Importantly, these abilities were not strongly related, suggesting that the cognitive processes underpinning understanding, naming, and facial selection operate independently and may deteriorate at different rates in AD.

**Conclusion:**

The findings highlight a nuanced interaction between emotional processing and cognitive functioning in AD. The ability to interpret emotional situations and associate them with specific facial expressions likely involves multiple domains of knowledge and neural networks, many of which are degraded in AD. This degradation contributes to higher odds of inaccuracies in emotional recognition and processing. These insights underscore the importance of considering both cognitive and emotional dimensions when evaluating social functioning in AD patients and tailoring interventions to preserve emotional and social competencies.

